# Rate and risk factors for AKI after CT scans in a cancer cohort 

**DOI:** 10.5414/CN109591

**Published:** 2018-11-12

**Authors:** Sheron Latcha, Andrew J. Plodkowski, Junting Zheng, Edgar A. Jaimes

**Affiliations:** 1 Renal Service,; 2 Department of Radiology, and; 3 Epidemiology and Biostatistics, Memorial Sloan Kettering Cancer Center,; 4 Weill Cornell Medical College, New York, NY, USA

**Keywords:** contrast nephropathy, nephrotoxicity, intravenous contrast, acute kidney injury rate, risk factors

## Abstract

Aims: The proinflammatory milieu in cancer patients may expose them to increased risk for acute kidney injury (AKI) after IV contrast (CON). The aims of this study were to determine: (1) the rates of AKI after CON and noncontrast (NC) CT scans in cancer inpatients, (2) if rates differed among cancer subtypes, and (3) whether recent chemotherapy, comorbid conditions, or nephrotoxins increase AKI after CON. Materials and methods: Retrospective data was collected on adults who had received a CON or NC CT from January 1, 2012 to December 30, 2014. AKI was defined as a > 1.5× increased baseline creatinine. Data was analyzed using Rao-Scott χ^2^-test, propensity score matching, and logistic regressions. Results: A total of 7,512 CT scans were performed in 4,456 patients (4,958 NC, 2,554 CON). The rate for AKI with CON was 7.3% and 11.4% (p < 0.001) with NC imaging. The risk of AKI increased with lower baseline eGFR: for eGFR ≤ 29 mL/min/1.73m^2^, OR = 1.83 (p = 0.0002); for eGFR 30 – 59 mL/min/1.73m^2^, OR = 1.5 compared to eGFR ≥ 60 mL/min/1.73m^2^ (p < 0.0001). AKI rates were higher when any chemotherapy was given within 60 days of CT (OR = 1.22, p < 0.02), with congestive heart failure (OR 1.51, p = 0.0006), and history of AKI (OR 3.89, p < 0.0001). In 1:1 propensity score matched samples, the OR for AKI after CON was 0.87 (p = 0.23) compared to NC. Conclusion: In cancer patients, eGFR below 59 mL/min/1.73m^2^ were associated with increased rate of AKI, independent of contrast exposure. Congestive heart failure and prior AKI were also associated with increased rates of AKI.

## Introduction 

Among cancer patients, understanding the frequency and risk factors for acute kidney injury (AKI) after IV contrast (CON) is particularly important since these patients rely on imaging studies with CON for diagnosis and staging of disease, to monitor response to treatment, and for surveillance for disease recurrence. There are few studies on AKI after CON in cancer patients. The majority of these reports are small and limited to critical care or Emergency Room populations [1, 2, 3]. Current data from noncancer cohorts suggests that lower estimated glomerular filtration rate (eGFRs), independent of CON, is the major risk factor for AKI. On the other hand, a recent observational study in a cancer cohort suggests that CON is indeed an independent risk factor for AKI [4]. This raises the possibility that AKI after CON may have different pathogeneses and frequencies among cancer and noncancer patients. 

Clinical and epidemiological studies suggest that dysregulation of inflammatory responses plays a role in the genesis and promotion of cancer growth [5]. Some of the same proinflammatory molecules involved in tumor-cell proliferation, transformation, and invasion have been implicated in the pathogenesis of AKI after CON. These include endothelin, reactive oxygen species, and hypoxia-inducible factor 1α [6, 7, 8, 9, 10, 11]. The available evidence suggests that cancer patients may have a higher rate of AKI from all causes. The odds ratio (OR) for AKI from all causes in cancer patients as compared to noncancer patients is 1.79 [12], and the reported 1- and 5-year risks for AKI in cancer patients are 17.5% and 27%, respectively, with corresponding risks of 6.7% and 18.6% in noncancer patients [13]. Whether cancer patients also have a higher rate of AKI after CON has not yet been investigated in a large cohort. 

To examine this, we designed a study to determine: (1) the rates of AKI after CON and noncontrast (NC) CT scans in cancer inpatients, (2) whether the rates of AKI differed among cancer subtypes, and (3) whether recent chemotherapy, comorbid conditions, or nephrotoxins are associated with AKI. 

## Material and methods 

After obtaining approval from the Institutional Review Board at Memorial Sloan Kettering Cancer, retrospective data was obtained from January 1, 2012 to December 30, 2014 on patients ≥ 18 years of age who had a CON or NC CT of the head, neck, or chest and who had a serum creatinine (SCr) within 3 days before and after CT. Because CT of the abdomen and pelvis are usually ordered with CON “by default”, and when they are not, the physician’s decision to not administer contrast may be influenced by the patient’s SCr, these studies were excluded to decrease bias. The same volume of CON is given for all CON CT scans. The dose of omnipaque300 (General Electric Healthcare, Princeton, NJ, USA) given for a SCr < 2 mg/dL is 75 – 100 cc. For a SCr ≥ 2 mg/dL, IV contrast is usually not given, and if it is, it is usually in consultation with a nephrologist. Exclusion criteria were: a CON CT ≤ 3 days before a NC CT, patients on either acute or chronic renal replacement therapy, and no matched pre- and post-CT SCr. If > 1 NC or CON CT was done on the same date, these were counted as one study. AKI was defined using the Kidney Disease Improving Global Outcomes criteria (increase in SCr 1.5× baseline). Urine output criteria were not used to define AKI. The baseline SCr was defined as the maximum SCr within 3 days prior to the CT scan. The eGFR was calculated using the 4-variable Modification of Diet in Renal Disease (MDRD) formula. International Statistical Classification of Diseases codes (ICD-9) were used to identify diagnoses and medications previously identified as risk factors for contrast-induced nephropathy (multiple myeloma (MM), diabetes mellitus (DM) congestive heart failure (CHF), hypotension, liver metastases, any chemotherapy given ≤ 60 days prior to CT, chronic kidney disease (CKD), nonsteroidal anti-inflammatory medications (NSAIDs), cyclooxygenase 2 inhibitors (COX2), angiotensin converting enzyme inhibitors (ACE I), angiotensin II receptor blockers (A2RB), vascular endothelial growth factor inhibitors (anti-VEGF), immune checkpoint inhibitors, tyrosine kinase inhibitors (TKI), gemcitabine, and cisplatin). Protected health information was coded in accordance with the requirements of the Health Insurance Portability and Accountability Act. 

### Statistical methods 

Associations between categorical variables, including AKI, CON/NC, patient characteristics, and other clinical variables were examined using the Rao-Scott χ^2^-test, considering that some patients had multiple scans during the study period. The continuous baseline eGFR was compared between patients with CON and NC as well as between patients with and without AKI, using generalized linear regressions with logit link function, and generalized estimating equations (GEE) with a robust covariance matrix and the exchangeable correlation structure, to take into account multiple scans in some patients. In a multivariable analysis, a generalized linear regression and the GEE method were used to assess associations between AKI and CON, controlling for other clinical factors univariately associated with AKI. Backward selection was used with a significance level at 0.05. OR of each covariate along with the 95% confidence interval were estimated in univariate and multivariable models. 

Propensity score matching was performed as a sensitivity analysis to minimize the confounding effects in this retrospective study. The propensity score was first calculated based on all variables univariately associated with CON. A 1:1 matching was then applied on propensity score allowing for a matching distance of 20% of the standard deviation. We reported standardized mean differences between CON and NC groups as a measure of balance in the matched data. The association between CON and the risk of AKI was examined using a conditional logistic regression. 

A test with p-value < 0.05 was considered statistically significant in our analyses. All statistical analyses were performed in software packages SAS 9.4 (SAS Institute Inc., Cary, NC, USA) and The R version 3.4 (R Foundation for Statistical Computing, Vienna, Austria). 

## Results 

A total of 7,512 CT scans were performed in 4,456 patients. There were 4,958 NC and 2,554 CON CT scans. Demographic data is shown in Table 1. The rate of AKI after CON was 7.3% and 11.4% in the NC group (p < 0.001). In both groups, AKI was associated with lower baseline eGFRs (Table 2). AKI rates at eGFRs ≤ 29 mL/min/1.73m^2^, 30 – 59 mL/min/1.73m^2^, and ≥ 60 mL/min/1.73m^2^ were 26.3%, 18%, and 7.5% in all scans; 13.3%, 11.5%, and 6.7% in the CON group and 26.9%, 20%, and 7.9% in the NC group, respectively. CON was ordered less frequently at lower eGFRs (4.4% at ≤ 29 mL/min/1.73m^2^, and 37.9% at ≥ 60 mL/min/1.73m^2^, p < 0.001). 

The initial observations are shown in Table 3. Significantly higher rates of AKI were observed in those treated with TKI/anti-VEGF, (p = 0.01), any chemotherapy within 60 days, CHF, CKD, AKI, brain and other nervous system tumors, leukemia, lymphoma, male genital system, myeloma, oral cavity and pharynx tumors, respiratory system tumors (all p < 0.001), digestive system and female genital system tumors (all p = 0.01). 

In multivariate analysis (Table 4) controlling for contrast, chemotherapy within 60 days (OR = 1.22, p = 0.02), CHF (OR = 1.51, p = 0.0006), prior AKI (OR = 3.89, p ≤ 0.0001), and lower eGFRs remained significant risk factors for AKI. The risk of AKI increased with lower baseline eGFR: for eGFR ≤ 29 mL/min/1.73m^2^, OR = 1.83 (p = 0.0002); for eGFR 30 – 59 mL/min/1.73m^2^, OR = 1.5 compared to eGFR ≥ 60 mL/min/1.73m^2^ (p < 0.0001). The risk of AKI was not different between CON and NC scans (OR = 1.08, 95% CI: 0.90 – 1.29, p = 0.42). None of the medications traditionally associated with AKI (NSAIDs, ACE I, A2RB) were found to be significantly associated with AKI. The rate of AKI for patients with history of DM, CKD, or hypotension was not different between the two groups. No cancer subtype was associated with a higher OR for AKI. 

To minimize confounding effects on contrast, we matched 2,257 pairs of CON and NC samples in 1:1 propensity score matching analysis. All variables were balanced in two sample groups (Table 5). The OR for AKI with contrast was 0.87 (95% CI: 0.87 – 1.09, p = 0.23). Realizing that the absolute numbers of patients in the propensity model at lower eGFRs were quite small and that it may be more meaningful to look at patients with a clearly abnormal creatinine at baseline or an eGFR < 45 mL/min/1.73m^2^, we performed multivariate analysis to look for associations with AKI in patients with eGFR < 45 mL/min/1.73m^2^. The multivariate analysis showed that the OR for AKI was still higher in the NC group, although not significant (OR 1.58, p = 0.17). 

## Discussion 

Recent large retrospective data base analyses in noncancer patients have brought into question whether contrast-induced nephropathy even exists, given that the reported rates of AKI following CON are similar to the basal rate of AKI observed in hospitalized patients [14, 15]. It has been assumed that the results from noncancer cohorts can be generalized to cancer patients. However, this population may have unique risk factors for AKI after CON, including exposure to tumoral cytokines, nephrotoxic chemotherapy, and complications from cancer treatment. This study reports the rate and risk factors for AKI after NC and CON CT scans in the largest cancer cohort examined so far. Unlike previous investigations, we did not exclude patients with low SCr values, and the AKI rate was measured using calculated eGFR as opposed to relying on ICD-9 coding. Consequently, we believe our study provides a better assessment of AKI after CT in a general cancer cohort. 

The rate of AKI after CON was 7.3%, significantly lower than the rate of AKI observed in patients who did not receive CON. Akin to data in noncancer cohorts [16, 17, 18], our analysis showed that AKI was significantly associated with overall lower baseline eGFR, independent of contrast exposure. Because there are ethical constraints to performing a randomized case control trial to examine the relationship between AKI and CON, especially at lower eGFRs, we used propensity score matching method to reach a 1:1 match in the CON and NC groups using all the variables that were associated with contrast. The propensity analysis showed a trend towards a lower rate of AKI in the CON group, but this did not reach statistical significance. In summary, none of the statistical models used in this analysis demonstrated a higher rate of AKI from CON exposure in cancer patients. 

Methodological issues make it difficult to make comparisons between findings from our study and those from studies done elsewhere in both cancer and noncancer cohorts. Our results do not support our supposition that the proinflammatory milieu of cancer results in an increased rate of AKI after CON. The observed rate of 7.3% is within the range of AKI after CON reported in noncancer cohorts [19]. Prior studies have reported higher rates of AKI in cancer patients after CON, ranging from 8 to 20% [1, 2, 3, 20]. However, these were smaller studies. 

The finding of a lower rate of AKI after CON exposure does not support any protective effect from its use, nor does it support the liberalization of current institution-specific or American College of Radiology screening guidelines [21] for prevention of contrast-induced nephropathy in patients with eGFRs ≤ 59 mL/min/1.73m^2^. While we tried to overcome some of the limitations inherent in analyses of retrospective data by utilizing propensity score modeling, the higher observed rate of AKI in the NC group in this report highlights a major limitation of retrospective data analysis. We were unable to ascertain whether patients with lower eGFRs received IV hydration with CON studies. Additionally, our data suggests that physicians may preferentially be ordering NC studies in patients with eGFRs ≤ 29 mL/min/1.73m^2^. In over 95% of cases, the physician ordered a NC CT for patients at this eGFR level. While our multivariate analysis showed that when controlling for comorbidities like prior AKI, CHF, and hypotension, the risk of AKI was not different between the CON and NC groups (p = 0.35), the possibility remains that clinical information that cannot be adequately captured from a retrospective data query played a role in the physicians’ decision to order a NC or a CON CT scan. 

Several groups of patients experienced significantly higher overall rates of AKI. One important and unique observation to cancer patients is that receiving any chemotherapy within ≤ 60 days of a CON study was associated with an increased risk for AKI. This observation has been made in other cancer cohorts [2, 4]. Therefore, patients on active or recent chemotherapy may require extra care and caution when ordering CON studies. Possible explanations for this finding include: (1) pre-existing prerenal azotemia or ischemic acute tubular necrosis from nausea, vomiting, and diarrhea; 2) direct nephrotoxicity of the cancer drugs and/or; 3) other nephrotoxic complications from treatment (tumor lysis syndrome, hypercalcemia). We found no correlation with any specific group of anticancer agent and AKI. As compared to previous observations [4], AKI rates were not significantly higher among patients with hematologic malignancies compared to those with solid tumors. 

A limitation of this and of all retrospective studies that have examined the relationship between AKI and CON is the relatively small number of patients with eGFRs ≤ 29 mL/min/1.73m^2^ who receive IV contrast. We used propensity score method to account for potentially-confounding variables, but these patients still accounted for < 1% of all CT studies. Limiting our data set to only those who had a CT of the head, neck, and chest did decrease the number of evaluable subjects, but since all contrast studies receive the same volume of contrast, this should not have significantly impacted the observations made. Future prospective studies utilizing a broader cancer cohort may still add new information to our current understanding of AKI after CON. 

## Conclusion 

This is the largest retrospective study to date that has examined the rate of AKI after NC and CON CT scans in a cancer cohort at a comprehensive cancer center. AKI was significantly associated with lower baseline eGFRs, independent of contrast exposure. Additional risk factors for AKI in cancer patients were the administration of chemotherapy within ≤ 60 days of CT, CHF, and prior AKI. The observed rate of AKI after IV contrast in a large cancer cohort was not appreciably different from prior reports on noncancer cohorts. 

## Funding 

This study was supported by Memorial Sloan Kettering Cancer Center Support grant/core grant P30CA008748 and Byrne Research Funds from the Department of Medicine at Memorial Sloan Kettering Cancer Center. 

## Conflict of interest 

None. 

**Table 1.** Patient characteristics at baseline.

	All scans	Noncontrast	Contrast
Gender			
Female	3,451 (45.94%)	2,151 (43.38%)	1,300 (50.9%)
Male	4,061 (54.06%)	2,807 (56.62%)	1,254 (49.1%)
Race			
Asian	519 (6.83%)	347 (7%)	172 (6.73%)
Black	677 (9.01%)	444 (8.96%)	233 (9.12%)
Native American	7 (0.09%)	5 (0.1%)	2 (0.08%)
White	6,309 (83.99%)	4,162 (83.95%)	2,147 (84.06%)
Ethnicity			
Hispanic or Latino	370 (4.93%)	240 (4.84%)	130 (5.09%)
No value entered	1 (0.01%)	0 (0%)	1 (0.04%)
Not Hispanic or Latino	7,141 (95.06%)	4,718 (95.16%)	2,423 (94.87%)
Malignancy type			
Bones and joints	139 (1.9%)	72 (1.5%)	67 (2.6%)
Brain and other nervous system	490 (6.5%)	388 (7.8%)	102 (4%)
Breast	471 (6.3%)	255 (5.1%)	216 (8.5%)
Digestive system	1,006 (13.4%)	511 (10.3%)	495 (19.4%)
Endocrine system	181 (2.4%)	103 (2.1%)	78 (3.1%)
Eye and orbit	24 (0.3%)	15 (0.3%)	9 (0.4%)
Female genital system	387 (5.2%)	130 (2.6%)	257 (10.1%)
Leukemia	2,033 (27.1%)	1,680 (33.9%)	353 (13.8%)
Lymphoma	1,620 (21.6%)	1,254 (25.3%)	366 (14.3%)
Male genital system	467 (6.2%)	311 (6.3%)	156 (6.1%)
Myeloma	425 (5.7%)	355 (7.2%)	70 (2.7%)
Oral cavity and pharynx	231 (3.1%)	88 (1.8%)	143 (5.6%)
Respiratory system	1,073 (14.3%)	561 (11.3%)	512 (20%)
Skin excluding basal and squamous	405 (5.4%)	291 (5.9%)	114 (4.5%)
Soft tissue including heart	281 (3.7%)	144 (2.9%)	137 (5.4%)
Urinary system	400 (5.3%)	244 (4.9%)	156 (6.1%)
Miscellaneous	4,012 (53.4%)	2,162 (43.6%)	1,850 (72.4%)

**Table 2.** Associations between age, gender, and eGFR with AKI and contrast.

	All scan (N = 7,513)	No AKI (n = 6,761)	AKI (n = 751)	p-value	AKI in noncontrast (N = 565)	AKI in contrast (N = 186)	p-value
Baseline eGFR, mean (SD) mL/min/1.73m^2^	96.6 (47.6)	98.3 (46.4)	81.4 (55.0)	< 0.001	72.6 (48.9)	108.1 (63.5)	< 0.001
CKD group				< 0.001			< 0.001
eGFR ≤ 29 mL/min/1.73m^2^	342 (4.6%)	252 (73.7%)	90 (26.3%)		88 (97.8%)	2 (2.2%)	
eGFR 30 – 59 mL/min/1.73m^2^	1,181 (15.7%)	968 (82%)	213 (18%)		182 (85.4%)	31 (14.6%)	
eGFR ≥ 60 mL/min/1.73m^2^	5,989 (79.7%)	5,541 (92.5%)	448 (7.5%)		295 (65.8%)	153 (34.2%)	
Age at CT, years				0.01			0.34
18 – 44	1,155 (15.4%)	1,055 (91.3%)	100 (8.7%)		74 (74%)	26 (26%)	
45 – 64	3,048 (40.6%)	2,755 (90.4%)	293 (9.6%)		224 (76.5%)	69 (23.5%)	
65 and above	3,309 (44%)	2,951 (89.2%)	358 (10.8%)		267 (74.6%)	91 (25.4%)	
Gender				< 0.001			< 0.001
Female	3,451 (45.9%)	3,153 (91.4%)	298 (8.6%)		211 (70.8%)	87 (29.2%)	
Male	4,061 (54.1%)	3,608 (88.8%)	453 (11.2%)		354 (78.1%)	99 (21.9%)	
Contrast				< 0.001			
No	4,958 (66%)	4,393 (88.6%)	565 (11.4%)				
Yes	2,554 (34%)	2,368 (92.7%)	186 (7.3%)				

CKD = chronic kidney disease; AKI = acute kidney injury; eGFR = estimated glomerular filtration rate.

**Table 3.** AKI by patient clinical factors at time of scan.

Factor	AKI (%)	No AKI (%)	p-value
Mutation status			
Any mutation	79 (10.5%)	793 (11.7%)	0.12
TKI/VEGF inhibitors	19 (2.5%)	261 (3.9%)	0.01
Immune checkpoint/BRAF/EGFR inhibitors	19 (2.5%)	171 (2.5%)	0.99
Chemotherapy within 60 days of CT scan			
Any chemotherapy	478 (63.6%)	3,769 (55.7%)	< 0.001
Bisphosphonates	1 (0.1%)	8 (0.1%)	0.92
Gemcitabine	27 (3.6%)	275 (4.1%)	0.31
Cisplatin	24 (3.2%)	177 (2.6%)	0.19
Medication			
ACE inhibitors	109 (14.5%)	1,063 (15.7%)	0.25
Angiotensin Receptor Blocker	69 (9.2%)	587 (8.7%)	0.49
NSAIDs and COX2 inhibitor	38 (5.1%)	458 (6.8%)	0.02
Comorbid disease			
Diabetes mellitus	207 (27.6%)	1,707 (25.2%)	0.07
Congestive heart failure	151 (20.1%)	684 (10.1%)	< 0.001
Hypotension	119 (15.8%)	1,131 (16.7%)	0.46
Liver metastasis	96 (12.8%)	1,208 (17.9%)	< 0.001
Chronic kidney disease	216 (28.8%)	893 (13.2%)	< 0.001
Acute kidney injury	510 (67.9%)	2,001 (29.6%)	< 0.001
Malignancy type			
Bones and joints	16 (2.1%)	123 (1.8%)	0.44
Brain and other nervous system	20 (2.7%)	470 (7%)	< 0.001
Breast	38 (5.1%)	433 (6.4%)	0.03
Digestive system	87 (11.6%)	919 (13.6%)	0.01
Endocrine system	13 (1.7%)	168 (2.5%)	0.07
Eye and orbit	2 (0.3%)	22 (0.3%)	0.79
Female genital system, in females	29 (3.9%)	358 (5.3%)	0.01
Leukemia	265 (35.3%)	1,768 (26.1%)	< 0.001
Lymphoma	202 (26.9%)	1,418 (21%)	< 0.001
Male genital system, in males	63 (8.4%)	404 (6%)	< 0.001
Myeloma	68 (9.1%)	357 (5.3%)	< 0.001
Oral cavity and pharynx	13 (1.7%)	218 (3.2%)	< 0.001
Respiratory system	84 (11.2%)	989 (14.6%)	< 0.001
Skin excluding basal and squamous	49 (6.5%)	356 (5.3%)	0.04
Soft tissue including heart	26 (3.5%)	255 (3.8%)	0.45
Urinary system	45 (6%)	355 (5.3%)	0.12

AKI = acute kidney injury; TKI = tyrosine kinase inhibitor; VEGF = vascular endothelial growth factor; BRAF = B-RAF kinase inhibitor; EGFR inhibitors = epidermal growth factor inhibitor; ACE inhibitors = angiotensin converting enzyme inhibitor; NSAIDs = non-steroidal anti-inflammatory drugs; COX2 inhibitor = cyclo oxygenase 2 inhibitor.

**Table 4.** Regression analysis on AKI in all scans.

	Univariate analysis	Multivariable analysis	
	OR of AKI (95%CI)	OR of AKI (95%CI)	p-value
Noncontrast (ref= contrast)	1.6 (1.34, 1.89)	1.08 (0.9, 1.29)	0.424
Baseline eGFR			
≤ 29 mL/min/1.73m^2^	4.01 (2.96, 5.43)	1.83 (1.33, 2.52)	0.001
30 – 59 mL/min/1.73m^2^	2.59 (2.13, 3.14)	1.5 (1.23, 1.84)	< 0.001
≥ 60 mL/min/1.73m^2^ (ref)			
Any chemotherapy	1.38 (1.17, 1.63)	1.22 (1.03, 1.45)	0.021
Brain and other nervous system malignancy	0.35 (0.21, 0.61)	0.57 (0.33, 0.96)	0.036
Congestive heart failure	2.32 (1.86, 2.89)	1.51 (1.19, 1.91)	0.001
Prior acute kidney injury	5.13 (4.32, 6.1)	3.89 (3.2, 4.73)	< 0.001

AKI = acute kidney injury; Ref = reference group; OR = odds ratio; eGFR = estimated glomerular filtration rate.

**Table 5.** Summary of propensity score-matched data.

	Noncontrast (n = 2,252)	Contrast (n = 2,252)	Standardized mean difference	Variance ratio
GFR group ( mL/min/1.73m^2^)				
GFR ≤ 29	17 (0.8%)	15 (0.7%)		Reference
GFR 30 – 59	273 (12.1%)	269 (11.9%)	–0.55	0.99
GFR ≥ 60	1962 (87.1%)	1,968 (87.4%)	0.8	0.98
Gender, male	1174 (52.1%)	1,154 (51.2%)	–1.78	1
Age (years)				
18 – 44	305 (13.5%)	322 (14.3%)		Reference
45 – 64	897 (39.8%)	915 (40.6%)	1.63	1.01
65 and above	1,050 (46.6%)	1,015 (45.1%)	–3.12	0.99
NSAIDs and COX2 inhibitors	171 (7.6%)	166 (7.4%)	–0.85	0.97
TKI and VEGF	92 (4.1%)	90 (4%)	–0.45	0.98
Any chemotherapy < 60 days	1,152 (51.2%)	1,172 (52%)	1.78	1
Tumor type				
Brain and other nervous system	96 (4.3%)	102 (4.5%)	1.28	1.06
Leukemia, lymphoma, and multiple myeloma	608 (27%)	610 (27.1%)	0.2	1
Genital	316 (14%)	304 (13.5%)	–1.56	0.97
Oral cavity and pharynx	76 (3.4%)	96 (4.3%)	4.4	1.25
Respiratory system	427 (19%)	425 (18.9%)	–0.23	1
Digestive system	403 (17.9%)	404 (17.9%)	0.12	1
Skin excluding basal and squamous	114 (5.1%)	114 (5.1%)	0	1
Comorbidities				
Diabetes mellitus	561 (24.9%)	548 (24.3%)	–1.34	0.98
Congestive heart failure	209 (9.3%)	207 (9.2%)	–0.31	0.99
Hypotension	306 (13.6%)	315 (14%)	1.15	1.02
Liver metastasis	532 (23.6%)	534 (23.7%)	0.21	1
Chronic kidney disease	186 (8.3%)	185 (8.2%)	–0.16	1
Acute kidney injury	538 (23.9%)	529 (23.5%)	–0.94	0.99

CKD = chronic kidney disease; GFR = glomerular filtration rate; NSAIDs = non-steroidal anti-inflammatory drugs; COX2 inhibitors = cyclo oxygenase 2 inhibitors; TKI = tyrosine kinase inhibitor; VEGF = vascular endothelial growth factor.
